# Home-Based Robotic Upper Limbs Cardiac Telerehabilitation System

**DOI:** 10.3390/ijerph191811628

**Published:** 2022-09-15

**Authors:** Bogdan Mocan, Mihaela Mocan, Mircea Fulea, Mircea Murar, Horea Feier

**Affiliations:** 1 Department of Design Engineering and Robotics, Technical University of Cluj-Napoca, 400020 Cluj-Napoca, Romania; 2 Department of Internal Medicine, University of Medicine and Pharmacy Iuliu Hatieganu Cluj-Napoca, 400012 Cluj-Napoca, Romania; 3 Institute for Cardiovascular Diseases Timisoara, University of Medicine and Pharmacy Timisoara, Gheorghe Adam Nr. 13A, 300310 Timisoara, Romania

**Keywords:** telerehabilitation, robotic system, patients management, online communication

## Abstract

This article proposes a new, improved home-based cardiac telerehabilitation system enhanced by a robotic and Virtual Reality module for cardiac patients to be used in their rehabilitation program. In this study, a novel strategy was used to integrate existing equipment and applications with newly developed ones, with the aim of reducing the need for technical skills of patients using remote control. Patients with acute or chronic heart diseases require long-term, individualized rehabilitation in order to promote their motor recovery and maintain an active and independent lifestyle. This will be accomplished by creating a system for at-home cardiac telerehabilitation augmented by a VR and cobot systems, which can be used long-term at home by each individual patient. In the pre-feasibility study carried out on healthy volunteers familiar with software applications and robotic systems, we demonstrate that RoboTeleRehab could be technically feasible both hardware and software.

## 1. Introduction

Both our everyday life and the health care systems have been significantly impacted by the COVID-19 outbreak. All non-urgent medical services were shut down during a global health emergency, requiring medical professionals to re-evaluate how they provide essential care. At the beginning of the COVID-19 pandemic, a number of cardiac rehabilitation (CR) facilities were closed in order to boost surge capacity and prevent COVID-19 infections among high-risk cardiovascular patients. Nevertheless, it is generally accepted that postponing CR is linked to poorer rates of program enrolment, attendance, and completion [1]. The European Association of Preventive Cardiology (EAPC) has issued a structured call to action in order to keep providing the crucial components of CR [2]. Cardiovascular patients’ rehabilitation requires continuous and long-term therapy. The target group for CR is well established by international guidelines and comprises patients with acute coronary syndromes within the last 12 months, symptomatic angina, peripheral artery disease, heart surgery for coronary bypass or valve replacement, chronic heart failure, and cardiac transplant [3,4,5,6]. In the USA, centers for telerehabilitation were approved during COVID-19 pandemic situation [7].

Additionally, the COVID-19 effect on hospitals throughout the world is leading to the early release of current patients, and both a halt to contact-reduction activities and a suspension of new patient admissions. For example, COVID-19 has made it harder for almost 2 million persons to access rehabilitation program in Europe alone [8,9].

By 2050, the World Health Organization predicts that the number of persons aged 65 and older would increase by 73% in industrialized nations and by 207% overall [9]. This age group is more susceptible to cardiovascular disease and needs CR to enhance their quality of life. Nonetheless, younger patients and patients who suffered of COVID-19 could benefit from home rehabilitation in the following years.

To lessen the strain on healthcare systems and offer cardiac patients a safe setting to continue their therapy, traditional rehabilitation programs need to be converted to telerehabilitation. Telerehabilitation is therapy that occurs outside of a hospital setting, primarily at home or in the community, and allows users to engage in a customized rehabilitation program [10]. The importance of telerehabilitation and home exercise is practically always emphasized in studies or reviews that are written in response to COVID-19’s physical treatment and rehabilitation needs [11], and many of these studies or reviews also offer guidelines for how to handle employee training, physician assessments, and discharge in such settings [12].

During cardiac telerehabilitation, a physical therapist or other healthcare practitioner directly engages with the patient, providing feedback and instructions via web interfaces. They can make required alterations to the exercise routine by assessing the patient’s progress. Furthermore, because to technical improvements over the last two decades, much work has gone into developing novel physical platforms, such as robotic and orthotic systems [2,13,14], and software platforms that integrate VR and AR exergames [15] to aid the telerehabilitation process and improve motor function recovery outcomes. Physical therapy is being replaced by the use of robots and robotic exoskeletons with haptic feedback or haptic aid [16] and augmented with VR or AR exergames for increasing motivation and adherence to rehabilitation therapy [15]. These technologies can be utilized to continue the rehabilitation process at home even if there is a COVID-19 or other pandemic situation outbreak. Furthermore, due to new preventative medical measures and the risk we expose them to, it is no longer possible to provide rehabilitation for the aged, regardless of pathology, in the same way that it was before the COVID-19 epidemic [8] The current global pandemic crisis is increasing the demand for robot-assisted rehabilitation (RAR) equipment to limit the danger of infection for both healthcare workers and patients.

Telerehabilitation therapies could be employed in phase II of the CR as well as in phase III of the CR to support long-term compliance with physical activity recommendations. The TELEREHAB III experiment [17,18] discovered that a 6-month telerehabilitation program resulted in increased physical fitness and quality of life (QoL). Furthermore, the experiment showed that this intervention (CR with telerehabilitation) resulted in long-term health benefits and was cost effective for up to two years. Motivating patients to accept and utilize a telerehabilitation system is essential to the success of such a system. 

Inspired by these ideas and associated challenges, we are confident that the development of a new home-based cardiac telerehabilitation system enhanced by a robotic and Virtual Reality (VR) module will bring multiple benefits to cardiac patients in their rehabilitation program. Thus, in this research, we outline the design and development of a new home-based cardiac telerehabilitation system enhanced by a robotic and Virtual Reality (VR) system consisting of (A) the patient’s home-based cardiac telerehabilitation system and (B) the clinician’s telemonitoring system. The proposed home-based cardiac telerehabilitation system (RoboTeleRehab) is intended to assist patients in regaining motor function following a severe cardiac event. During unsupervised therapy at home, it is difficult for the patient to maintain motivation for therapeutic compliance. A combination of a VR module and a collaborative robot (cobot) can provide the required incentive by making the exercises safer, more comfortable, more appealing, and more enjoyable. This helps the therapist create rigorous, repeating training sessions for the patients and make them more enjoyable, engaging, and motivating. VR-based therapy can boost patient engagement by incorporating interactive and competitive challenges with frequent performance feedback throughout the exercise.

## 2. Materials and Methods

### 2.1. Robots as Exercise Devices for Upper Limb

Robotic devices are becoming increasingly popular for use in rehabilitation after a major cardiac event including open heart surgery as exercise devices for upper limb. The basic idea being implemented in different approaches [19,20,21] to facilitate rehabilitation is to have the robot make physical contact with the user’s upper limbs. Robotic therapy has been created to replicate precise actions repeatedly with minimal physical effort and time from therapists. Much of the recent research has been on creating increasingly complex robotic systems with a high number of DoFs to aid in the training of more complex movements like multi-joint arm and hand movements [22]. The development of control techniques, which outline how these technologies interact with people, has advanced beyond the robotic system. Robotic therapy control algorithms are designed to coordinate the usage of robotic systems for therapeutic exercise in a way that the participant’s selected activities promote motor plasticity and so improve motor recovery [23]. However, there is still little consensus among scientists about the most effective means of achieving this objective. As a result, various robot structures and control algorithms for robotic treatment have been developed on an as-needed basis, often utilizing ideas from the literature on rehabilitation, neurology, and motor learning [24]. The assistive control paradigm is the most advanced. The use of assistive controllers, which are analogous to “active assist” exercises carried out by rehabilitation therapists, enables participants to move their weaker upper limbs in the appropriate ways. Control strategies that provide resistance to the person that moves their limbs while exercising, demand a certain way to generate the needed force, or amplify movement faults are all examples of such devices (“error amplification” strategies). 

The RoboTeleRehab system objective is to give “support-as-needed” for the rehabilitation patient, which refers to help the participant as much as is required to complete the job; this means both providing support and resistance during recovery exercises. 

### 2.2. Design the Homebased Telerehabilitation Robotic System—RoboTeleRehab

To have a good QoL [25], following an acute event, all cardiac patients should be engaged right away in a rehabilitation program. A proper CR is required for these people’s freedom to live their lives as they see fit and to live better. It has been demonstrated [18] that exercise ability, as evaluated by peak oxygen consumption, exerts a significant long-term influence on prognosis following coronary artery bypass grafting. Utilizing a robotic telerehabilitation device to conduct a comprehensive cardiac rehabilitation program can facilitate home-based rehabilitation greatly. Most of these rehabilitation therapies require motor rehabilitation sessions to increase joint mobility, increase the heart’s ability to pump blood, and remove restrictions. This is the point where robotic systems outperform humans, which prompted us to initiate two research [22] to identify the various robotic structures utilized in CR.

**Figure 1:**
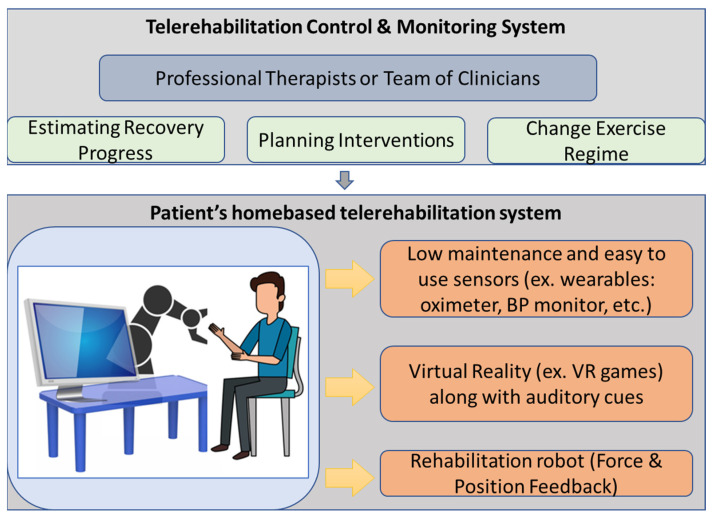
RoboTeleRehab system overview.

Starting from the research carried out in the field of robot-assisted rehabilitation and VR-based therapy and having in mind the need for such alternative models of delivery the CR therapy, RoboTeleRehab system (Figure 1) aims to provide a new home-based cardiac telerehabilitation system augmented by a robotic and VR module. Furthermore, we defined four requirements to be taken into consideration when developing the solution: safe, easy to use, easy to communicate, and affordable. Affordable price in the field of cardiac rehabilitation, meaning several tens of thousands of euros.

Thus resulted RoboTeleRehab, which is composed of the following: (A) patient’s home-based telerehabilitation system and (B) clinician’s remote and telemonitoring system. Figure 1 provides an overview of the RoboTeleRehab system presented in this paper.

The home-based telerehabilitation system (A) for the patient consist of:

▪ (A.1) A compact, lightweight cobot developed for direct human–machine interaction with six degrees of freedom (DoF)—used to support the patient arm motion during cardiac rehabilitation therapy; the cobot will be for medical applications based on sensitivity—it will be sensitive and secure due to the installation of force torque sensors in each of the seven axes; therefore, it is appropriate to be integrated into a rehabilitation system.
▪ (A.2) Participants’ arms may be positioned in a wide range of motion in three dimensions thanks to the robot controller’s intrinsic compliance possibilities.
▪ (A.3) PLC controller facilitating the control and connection between robot and VR platform.
▪ (A.4) High-end PC which runs the VR application.
▪ (A.5) An Android tablet for collecting the vital signs from the Bluetooth biosensors (blood pressure meter, ECG, oximeter).
▪ (A.6) Sensors connected to telerehabilitation system—e.g., blood pressure meter oximeter and ECG.

As it is highlighted above, the RoboTeleRehab system integrates a robot—a collaborative robot. In contrast to industrial robots, which are not designed to operate with humans, collaborative robots are created to coexist peacefully within the same work envelope. While collaborative robots may certainly improve workplace safety, it is a prevalent misconception that they are risk-free once implemented. Usage of power and force limits is a common approach to ensuring a safe environment for collaborative robots to work in. In this case, the danger of damages to a human is greatly decreased since a collaborative robot is limited in its ability to use maximum strength and force [26]. In addition, modern collaborative robots have a complex system of sensors (e.g., force sensors) that cause them to slow down when a human worker apply a force and to stop functioning entirely if the human increase the applied force above a certain threshold. Making them easier to program is another benefit for safety [27]. The low weight of the collaborative robot also represents a safety element. All these safety characteristics made it a candidate for integration within the RoboTeleRehab rehabilitation system. In a future article, a risk analysis will be made based on the ISO/TS 15066 standard to identify all mechanical, electrical, functional, and combined hazards in order to evaluate the risk associated with RoboTeleRehab system and to find solutions for their elimination. The Doctor’s control and telemonitoring system (B) will consist of the following:

▪ (B.1) A computer on which the data of up to six patients can be displayed and analyzed.
▪ (B.2) Software platform adopting the IoT paradigm and OSGI middleware enabling and facilitating VR platform, communication, monitoring, and remote control of the robotic system [28].

**Figure 2:**
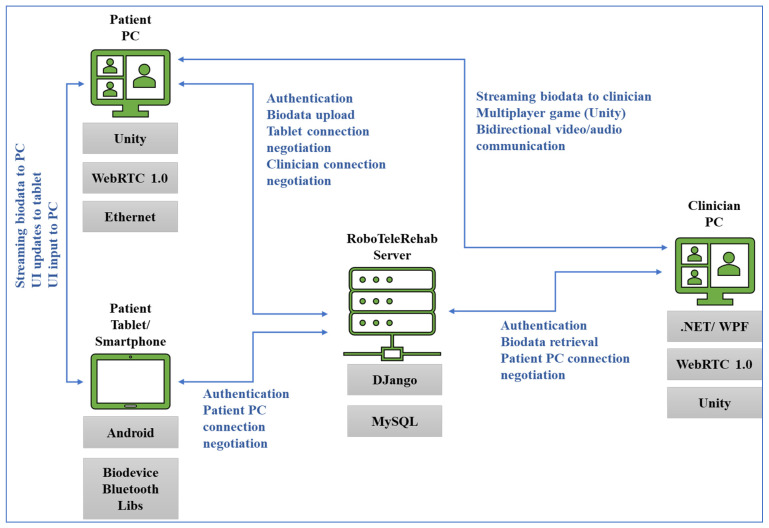
RoboTeleRehab system dataflow.

The patient’s home-based telerehabilitation system and clinician’s telemonitoring system will be coherently integrated within a cardio home-based telerehabilitation system. Furthermore, while the patients will use this telerehabilitation system, they will interact with the VR Exergame and professional therapist, which should increase the patient confidence and well-being, like in Figure 2 where the RoboTeleRehab system dataflow is highlighted.

### 2.3. Develop the Control Strategy of the RoboTeleRehab—Patient System

**Figure 3:**
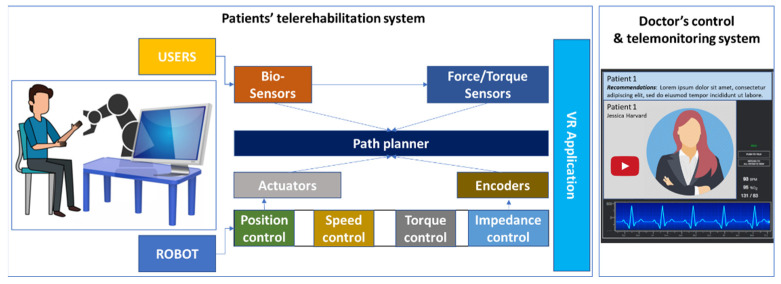
RoboTeleRehab control strategy—robotic system control loop.

In developing the control (Figure 3) of the RoboTeleRehab robotic system, we focused on improving the control and interaction between the patient and the telerehabilitation system using bio-signals, i.e., blood pressure meter, ECG, and oximeter. During movements, the RoboTeleRehab control strategy can be optimized using sensors information and dynamic control models to compensate for dynamic and nonlinear friction effects. Predicting the movement of a subject outside a resting position is impossible only by analyzing the operator’s behavior or interaction with the system without a bio-sensory system. The control strategy that will be implemented throughout the RoboTeleRehab system, so as to facilitate robot–patient collaboration, is intended to increase the level of patient participation and patient motivation. 

The robot will demonstrate the appropriate dynamic behavior, and its movements will depend on the forces that patients’ interactions exert. Moreover, the patient’s motivation will be done through a virtual reality system, which will allow for the therapist to establish a personalized rehabilitation therapy.

A Siemens controller that merges the capabilities of a PC-based platform that is already set up with Windows with programmable logic controllers that is open and safe will serve as the main control component of the RoboTeleRehab system. The RoboTeleRehab system’s primary control element is IoT Enterprise 10. Using the Simatic ODK 1500 software controller operating on open controller hardware, modern programming languages may be smoothly integrated and coupled with open controller hardware resources. Simulink and virtual reality applications are consequently set up and running on the open controller. A CANopen communication module is used to distribute the data obtained from Simulink to each robot joint actuator controller once it has been converted to the open controller hardware.

A control button is used to select the mode of operation. The assisted, partially assisted, and resistive are being developed as rehabilitation strategies for cardiac patients. Without the operator having to spend much effort, the robot drive systems move the subject’s arms utilizing the robot structure in assisted mode. This is done based on the movement parameters provided by Simulink in conjunction to the chosen rehabilitation activity. In a partially assisted mode of operation, the motors only provide a limited amount of torque to counteract the subject’s movements in order to achieve the target position while the Simulink algorithm takes into account sensor data. In the resistive mode of operation, the operating system of the robot opposes the subject’s motions to increase the amount of effort required for the subject to complete a certain job. Using the joystick buttons, patients may adjust the robot drive system’s working speed and torque during rehabilitation exercises in each mode of operation.

The foundation of the robot control loop in Figure 3 is a three-level cascading PI(D) loop, which is typical for motion control systems. The common drive’s location, acceleration, and velocity are input into the main control loop by the route planner, which then controls the common drive’s motion and outputs the acceleration and velocity. The speed control reference current for the actuator’s innermost loop is produced by the inner loop. The robot’s motor encoders provide the speed and position control loops with the necessary data.

In addition to the default safety systems of the collaborative robot, its final effector will be equipped with an emergency stop button (dead man switch), which will facilitate its stopping by the patient.

### 2.4. Design the VR Module for Patient’s Homebased Telerehabilitation System

The patient will be immersed in a 3D computer-generated environment with trees, flying objects, and other patient avatars, all generated by the VR module developed using Unity3D game engine. To incorporate RoboTeleRehab data (force from the robot force sensor) into the Unity3D environment, new classes of objects will be created that control the patient rehabilitation system.

One may change the virtual camera’s position and angle to highlight the patient’s motion using a six-axis force sensor and TCP monitoring. The patient mostly makes use of the tablet to log in, choose and alter menu items, and control the robot training scenarios settings. A system that creates the Unity3D environment in coordination with the robot force sensor and the training scenarios that the clinician has chosen is the high-end graphic control PC. This computer system, which also functions as a data transmission hub, renders the Unity3D environment, transmits medical data to the patient’s data using a standard encryption mechanism, and saves patient data to a cloud-based server using a Django application and a SQL database (Figure 2).

**Figure 4:**
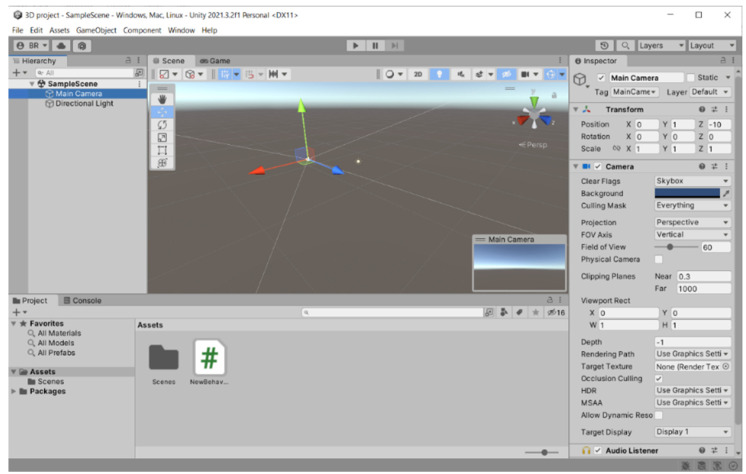
VR module (Unity3D capture).

In addition to the audio–visual link (through WebRTC) between each patient and the doctor, the multi-player features of Unity3D allow for each patient to see the avatars of the other patients. Each patient gets access to fully animated avatars, enabling interaction and exercise from the patient. The system was based on multi-user capabilities of Unity3D. A central cloud-based gaming engine (Figure 4) that allows registered players to pursue moving targets or other players and earn points based on their physical performance as determined by wireless EKG sensors, oximeters, and blood pressure monitors is connected to a maximum of six patients through the internet.

Players must find certain objects like spheres that, if collided with, will result in the accumulation of points in the virtual world’s hilly landscape (Figure 4). The player with the best score and the finest physiological state as determined by wearable medical sensors will win the game.

### 2.5. Design the Telemonitoring System for Doctors

A PC monitoring center and a mobile client will be part of the software for the clinician telemonitoring system, which will be created utilizing Java Web, PhoneGap technology, and a SQL Server database (Figure 2). The fundamental components of system design and development include the use of database-connecting tools like JavaBean, JDBC, and Hibernate, as well as database sheet design, validation of related database sheet attributes, implementation of core code and coding, data processing for dynamic pages, and utilization of the Bootstrap framework. Meteor will be used for data collection, transmission, and developing a single-chip microprocessor control software.

### 2.6. Testing Strategy of the Homebased Telerehabilitation System Prototype Enhanced with a Virtual Reality Exergame—RoboTeleRehab 

The RoboTeleRehab system validation experiments will take place in two stages, as stated in Reference [26]: the first stage will take place on healthy volunteers and the second stage will be on cardiac patients. Both stages of experiments will be conducted at Emergency County Hospital Cluj-Napoca, a teaching hospital linked with a university, which is a tertiary care facility. In the case of stage one most of the individuals that are recruited will be research lab stuff. The Iuliu Hatieganu University of Medicine and Pharmacy’s Ethics Committee (reg. no. 350/2.10.2019) will grant the study ethical permission. Each volunteer will sign a permission after being fully briefed. 

The following prerequisites must be fulfilled by research participants be able to understand and sign an informed consent form; be at least 18 years old; and be free of any joint, muscular, cardiac, or vascular disorders. If a participant meets any of the following requirements, they are disqualified for the trial: poor understanding of the Romanian language; recent upper-limb injuries.

The RoboTeleRehab will be taught to all the participants. Each individual test will last around 20 min. For the volunteer group, one of the medical staff will supervise the training. A satisfaction questionnaire (a modified form of Telerehabilitation Questionnaire) will be used [29].

For the patient group, two dedicated, unblinded employees with prior training in cardiovascular rehabilitation will conduct the training. Optitrack V100:R2 cameras will be used to record the motions. EMG signals will be captured utilizing a wireless system (such Delsys TrignoTM Delsys Inc., Boston, MA, USA) to measure the muscle movement. The duration of the workout as well as the quantity of repetition cycles will be recorded. Throughout the activities, cardiac frequency and SaO_2_ will be observed and noted. Feedback from the volunteer regarding pressure or discomfort will be continuously recorded. Throughout the test, the pain level will be assessed using a numerical rating scale every five minutes. After the exam, the Short Form McGill Pain Questionnaire, version 2, will be used to assess the degree of pain.

To adjust the RoboTeleRehab system as needed, the collected data will be registered into a database for descriptive and statistic evaluation. The technical design team will get a final report, which will then be debated. The technical team will improve the RoboTeleRehab structure and control based on the findings of the laboratory, volunteer, and cardiac patient testing.

## 3. Results

### 3.1. RoboTeleRehab System Overview

In order to meet the requirements of the advised CR, the RoboTeleRehab system was developed to train many upper-limb joints in a continuous 3D workspace. A commercially available cobot with two force sensors (six-axis force sensors SRI M3715C and SRI M3713C), and a custom handlebar make up the system’s major parts. The cobot is comprised by one robot arm, one robot controller, a touchpad, and a pre-installed application called “PolyScope”. The cobot arm is a 6-DoF kinematic equipment that could satisfy all the mobility criteria of a human arm rehabilitation (5 DoFs) [30]. Accessing the cobot arm’s joint velocities and locations in real time is a breeze with the help of the robot’s in-built features. A six-argument vector (X, Y, Z, Rx, Ry, and Rz) defines the position of the tool center point (TCP) relative to the UR arm’s base. The TCP’s location in three-dimensional space is denoted by the X, Y, and Z coordinates, while its rotation is indicated by the Rx, Ry, and Rz coordinates. In order to track the forces that patients and robots are subjected to in real time, a force sensor will be inserted between the TCP and the robot’s newly constructed handlebar. 

Cardiac patients following an open-heart surgery can use the modified handlebar since it also provides a support for the patients’ arm and Velcro^©^ straps to secure the hands to the grip. If the robot’s motion or speed veers outside of set parameters, it will halt. Operators can also stop the robot at any time by using the stop command and the emergency button. The robot will be able to talk with a PC through a router and transmit and receive orders and feedback using the Modbus TCP/IP protocol. Using a Bluetooth-enabled Android tablet, the blood pressure meter, oximeter, and ECG sensors in the system will be used to collect data (Figure 2). The patient picks and alters menu items, maintains the statuses of the robot system, and starts authentication using the tablet, which acts as the primary interface. The Human–Machine Interface (HMI) control PC is a system that creates the Unity3D environment in line with the robot sensors and the exercises characteristics provided by the professional rehabilitation staff. Figure 2 shows how this computer system will be used as a data transmission HUB and be responsible for carrying out the following tasks: establishing a secure audio/video link between the patient and the clinician; performing medical data transmission; rendering the Unity3D world based on robot sensor data; controlling the robot rehabilitation mode (passive-active-reactive), using the VR generated world and resistance limits specified by the clinician; and communicating with other computers.

### 3.2. RoboTeleRehab System for Patient

**Figure 5:**
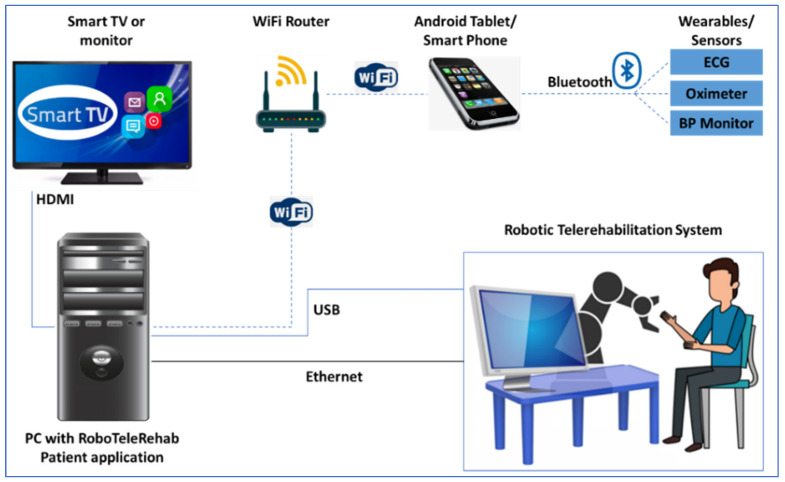
RoboTeleRehab patient hardware.

Figure 5 highlights the hardware setup of the patient’s home-based telerehabilitation equipment. 

**Figure 6:**
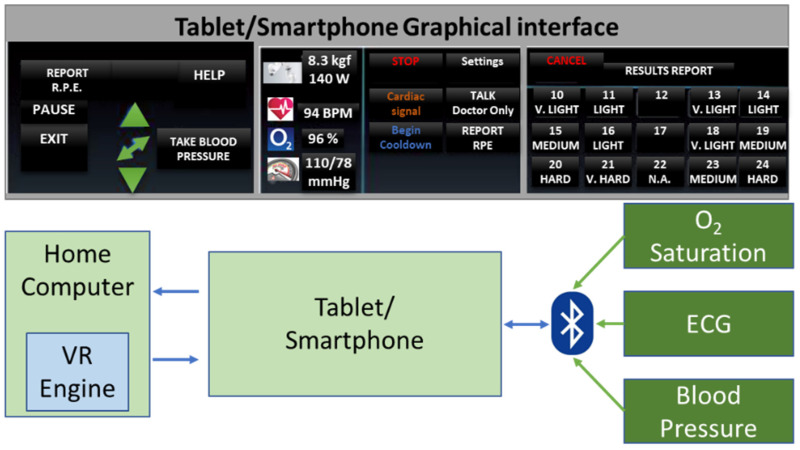
Tablet/smartphone interface and data HUB.

A collaborative robot (cobot) is part of the system, and it is mounted on a pedestal and linked to the patient’s PC via an ethernet interface and the TCP/IP protocol. For safety reasons, the robot actuation system can only be used if the emergency stop button has not been activated. The end effector has a stop button installed/dead-man switch, and the exoskeleton’s base structure also has a button installed in case of an emergency. If an emergency occurs, the patient must use the stop button. Once the dead-man switch is in the middle position, the robotic system will obey the motion commands input in Simulink. Actuators are required to stop working if the dead-man switch is disengaged or pressed too forcefully. The device will also come with an Android tablet that uses Bluetooth to sync the ECG, oximeter, and blood pressure sensors (Figure 6). The patient starts authentication, chooses, and updates menu items, and manages robot system statuses all using the tablet as their primary interface. A Unity3D world is rendered by the high-end graphic control PC in time with the robot sensors and the training environments chosen by the rehabilitation specialist.

The system will act as a hub for data transfer (Figure 6); thus, it will:

▪ Generate the Unity3D environment using information from robot sensors.
▪ Control the robot’s rehabilitation mode (assistive, passive, resistive) using the VR-generated world and resistance limitations (resistive force) established by the doctor.
▪ Create a secure link (audio/video) between the patient and doctor using WebRTC.
▪ Transfer data (patient medical data) while utilizing a common encryption method.
▪ Utilizing the Django application and the MySQL database, the patient data will be saved to a cloud server.
▪ Receive doctor data that is encrypted outlining the minimal and maximal (assistive or resistive) power force a patient should experience from the robot during training.
▪ If the system is set to multiplayer, update the locations and conditions of other patients’ avatars through the internet.

### 3.3. RoboTeleRehab System for Doctors

**Figure 7:**
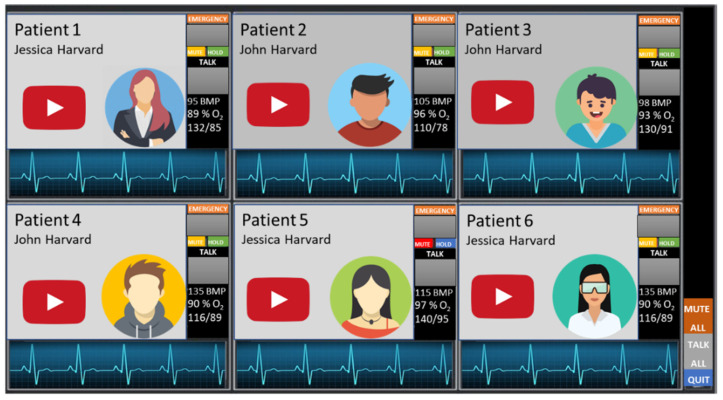
Demo design for physician HMI, which shows six patients in a window.

**Figure 8:**
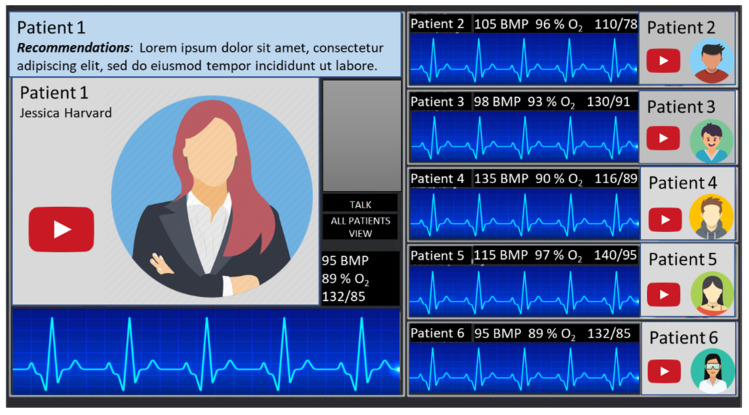
Demo design for physician HMI that focuses on a single patient.

On the physicians’ side, the system is constructed on a powerful computer that can display and analyze data from up to six patients (Figure 7 and Figure 8). The session will commence by first authenticating the patient and physician with the server for RoboTeleRehab system. Each patient is given an encryption key after the connection has been made, and this key is used to encrypt all data sent throughout the system during a session. The healthcare professional develops a special exercise plan for each patient using an exercise program editor. There might be any number of phases with different durations and power output objectives in each patient-specific exercise plan. A warm-up, the exercise, and a cool-down phase are usually included in these workout regimens.

The exercise routines provide both global and per-stage details (Figure 7). Global values indicate that these values are applicable to all phases of the workout prescription:

▪ Day goal(s): the suggested amount of exercise sessions per day for a patient.
▪ Weekly goal(s): the recommended number of workouts each week.
▪ Oxygen saturation: the oxygen saturation minimal value.
▪ Perceived effort (PE): the level of perceived effort.
▪ Perceived discomfort (PD): the level of perceived discomfort.

Per-exercise values indicate that these values define the various exercise of the prescribed workout. It is possible to add, remove, and rearrange stages. Each phase includes the functions listed below:

▪ Exercise Name: the exercise and phase name (e.g., warm-up, cardiovascular training).
▪ Exercise Length: minutes and seconds that the workout lasted.
▪ Target Heart Rate: specify the patient’s lowest and maximum heart rate targets.
▪ The target effort: in Watts for this exercise. These parameters are utilized to modify the mechanical resistance of the patient’s rehabilitation exercises (s). If the patient’s input force requirements are high, more robot resistance will be made available for them to work against in order to enhance their output of effort.
▪ PE Prompts: the device can alert patients automatically for PE at predefined intervals.
▪ PD evaluation: the patient can stop the exercise any time due to the increased level of discomfort.
▪ Blood pressure (BP) measurements: after a particular % of the stage has been completed, the device can automatically launch a blood pressure measurement.
▪ Planned date for rehabilitation session: each patient has a calendar that help him/her to plan and track the rehabilitation sessions.

For patients who have completed their rehabilitation sessions, RoboTeleRehab creates a report detailing their clinical data, like heart rate values (Min/Avg/Max), ECG, blood pressure (mmHg), RPE peak value, and percent of blood oxygen saturation at the beginning, during, and after the exercise. 

Privacy and data integrity of the patients: Data integrity and patient confidentiality are protected by RoboTeleRehab. Given that RoboTeleRehab will use public networks, this is crucial. Data about patients will be protected via RoboTeleRehab in two ways: Using HTTPS encryption, data are sent securely data server cloud based. This makes it possible for the patient and doctor to swap encryption keys at the start of each session. The communication between RoboTeleRehab and the cloud-based data server is shielded from port scanning via a virtual peripheral network (VPN). The doctor can reliably monitor each patient after setting up encrypted connections with them. Professionals have safe access to the patient’s medical records because the system is used through the hospital’s intranet. To create tailored exercise programs for each patient depending on their physical condition, the doctor requires access to the patient’s medical records. The system may inspect the biosensor values during a session and determine whether they are within acceptable bounds in order to confirm data integrity. Additionally, the integrity protocols included in the encryption used to encrypt the data, AES-GCM, may be utilized to identify transmission flaws in the data sent from patient to clinician. The system depends on the HTTPS validation procedure in order to ensure the integrity of data uploaded to the cloud-based server. The typical bandwidth of a Romanian home networking is at least 20 Mbps uploads/downloads. With a video resolution of 1920 × 1200 pixels, WebRTC consumes most of the bandwidth for the RoboTeleRehab system. Supplementary bandwidth is required every minute to upload data to the cloud-based server. The largest files are the ECG data files, each measuring 500 kB (sampling rate of ECG procedure). RoboTeleRehab requires at least 5 Mbps up/down for all data transfers, which is well within the capacity of home networking in terms of overall bandwidth

**Figure 9:**
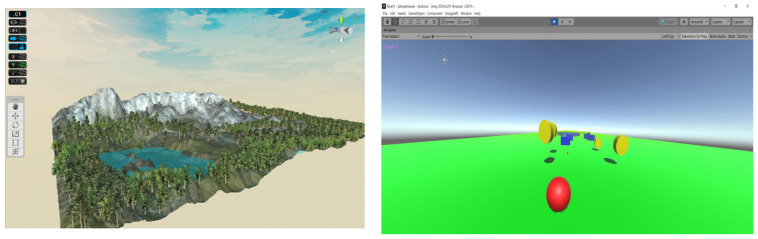
The VR application module to encourage adherence to the rehabilitation process.

The Unity3D game engine was utilized in the VR application to promote adherence to the rehabilitation process and to present a virtual world to the patient made up of things like landscapes, trees, moving spheres, and avatars of other patients. This system will encourage cardiac patients to interact with virtual objects that appear in the scene (Figure 9). 

The RoboTeleRehab VR platform is a versatile, inexpensive, and non-immersive VR system that replicates VR features using off-the-shelf gadgets like the OpenCV AI Kit OAK—1 (OAK—1 is a 4K camera capable of running the cutting-edge neural networks TensorFlow). The integrated robotic system tracks the upper body’s range of motion, gestures, and postural control and balance.

### 3.4. Prefesability Study of the Homebased Telerehabilitation System Prototype Augmented with a Robot and Virtual Reality Module—RoboTeleRehab 

**Figure 10:**
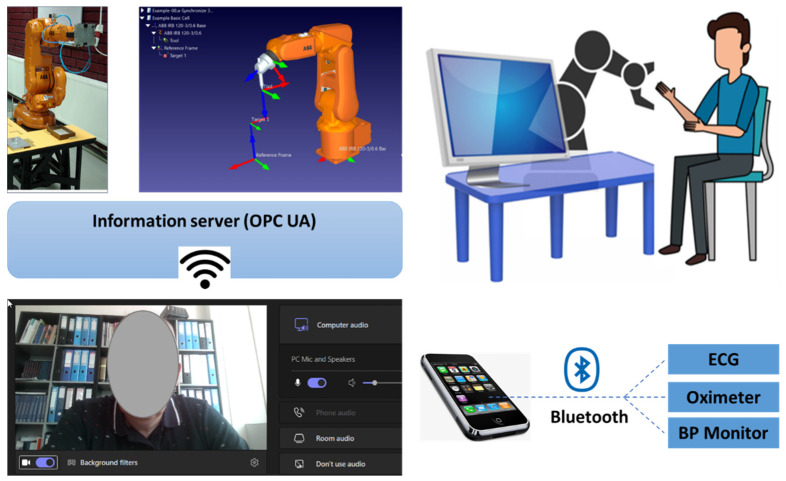
Lab testing of the robotic system for upper limb mobilization.

To demonstrate the feasibility of the idea and telerehabilitation system, we ran into a series of lab tests using the ABB IRB 140 robotic system and developed software application RoboTeleRehab (Figure 10). The tests were done on six volunteers from the Robotisation Manufacturing lab within the Technical University of Cluj-Napoca, who were familiar with robotic systems and video conferencing software applications.

Briefly stated, the goal of this robotic system (RoboTeleRehab) was to create a research prototype that can provide cardiac patients more motor performance gains for upper limb than typical manual therapy and other rehabilitation strategies. One ABB IRB 140 industrial robot, one JR3 force/torque sensor installed on the robot flange, one PC, and one network switch made up the lab testing system. The ABB IRB 140 was a six DoF motor-driven robot arm with a payload of 5 kg. It was based on a force sensor, which ensured compliance during movements and allowed participants’ arms to be positioned within a wide range of motion in three dimensions. Additionally, a number of safety measures were added to both the hardware and the software. Six participants (students) take part in the lab experiment to evaluate the feasibility of the telerehabilitation system. Force was measured by the force sensor at 2000 Hz during robot training procedures, and then down sampled at 50 Hz to match moving trajectories generated by robot system. It should be noted that the force measured was the interaction force between the participant’s limbs and the robot, not the net joint force. The Creaform C-Track 780 collected elbow and shoulder angle data both with and without robot training. 

The test was carried out like this: The exercise was established by consulting specialist doctors. In order to allow remote control of the robot from the therapist’s side, the physical therapist first phoned the patient through MS Teams at the prearranged time and gave them instructions on how a robot’s interface may be connected to and controlled remotely. For this, two interfaces were developed; the first is a Python-based advanced control interface, and the second is a Node-RED-based interface that enables mobile device control of the robot by the operator. The interface can read and write data on the server after establishing a connection with it.

In order to test the proposed system, we recruited six volunteers to form our research lab who agreed to participate to the study by signing a informed consent form. The detailed general and medical characteristics are summarized in Table 1. The workout was then started by one of our colleagues by launching the app for regulating the pulse oximeter. A VR workout program was then carried out after checking the volunteers’ vital signs and basic motor skills. The training was short and consisted of a printed page with the exercise program and the verbal explanation on how to coordinate VR with the movements of the upper arm. The session lasted 20 min in total, including preparatory time. 

The volunteers were supervised by our physician during the session. Feedback from the volunteer regarding pressure or discomfort was continuously recorded. The acceptance level was monitored using a modified form of Telerehabilitation Questionnaire, introduced in a Google form [2]. The results of the satisfaction questionnaire were synthetized in Table 2.

Overall, the subjects were satisfied with using the telerehabilitation system. The VR proved to be well accepted and telecommunication during exercises very easy. The usability test findings showed that users were happy and enthusiastic about the way they have interacted with the RoboTeleRehab system.

## 4. Discussion

In this paper, we discussed the design and the preliminary implementation of a new home-based cardiac telerehabilitation system enhanced by a robotic and Virtual Reality (VR) systems—called RoboTeleRehab. This work was based on our earlier efforts highlighted within the references [1,2], and it represents the expansion of cardiac patients’ assisted rehabilitation from specialist facilities and hospitals to home-based centers.

RoboTeleRehab system (Figure 1) aims to provide a new home-based cardiac telerehabilitation system augmented by a robotic and VR module composed of: (A) the patient’s home-based telerehabilitation system and (B) clinician’s telemonitoring system. The patient home-based telerehabilitation system consists of a commercially available cobot with two force sensors (six-axis force sensors SRI M3715C and SRI M3713C) and a custom handlebar. The cobot is composed by one robot arm, one robot controller, a touchpad, and a pre-installed application called “PolyScope.” The cobot arm is a 6-DoF kinematic equipment that could satisfy all the mobility criteria of a human arm rehabilitation (5 DoFs) [31]. Accessing the cobot arm’s joint velocities and locations in real time is a breeze with the help of the robot’s in-built features [32].

Patients with orthopedic, neurological, and pulmonary disorders as well as the neurosurgical patients have benefited from a variety of telerehabilitation programs, which have been proven to be beneficial [33]. Telerehabilitation is thought to provide patients possible advantages such as improved accessibility, less time commitment, and cost efficiency [34,35]. Additionally, research suggests that it can be just as successful as on-site rehabilitation [36]. Rare reports of telerehabilitation systems being used with patients following a major cardiac event exist, but the pandemic and COVID-19 infection have stoked increased interest in this field. A telerehabilitation system for cardiac patients placed in isolation certainly need different capabilities than earlier telerehabilitation systems did.

In this work, we first defined four requirements: safe, easy to use, easy to communicate, and affordable as essential for the new RoboTeleRehab system. Based on those prerequisites, we begin to design the solution. Easy to use is a critical component of software applications [37], but it plays a crucial role also in telerehabilitation for cardiac patients [12]. The user must manage the cobot and programs as well as carry out exercise without any on-site support, only by online supervision. Therefore, to properly execute the exercise sessions with patients who have varying degrees of computer literacy, a high level of usability is required. While they could clash with easy to use, safety, easy to communicate, and affordability were equally crucial. Making a system that is specifically designed for the cardiac patients, for instance, is the most effective technique to make the telerehabilitation system user-friendly. The creation of personalized systems, however, may make them less affordable. Additionally, the system would become more complex because monitoring is required for patient safety. In this study, a novel strategy was used to integrate existing equipment and applications with newly developed ones, with the aim of reducing the need for technical skills of patients and eventually use remote control. The RoboTeleRehab system was designed so that the robot control is local, the program for the robot will be selected locally by the patient upon the indication of the specialist (the programs for the robot can be transferred via the Internet), and the reaching of the target by the robot TCP will be done in accordance with the learned position and in correlation with the force applied by the patient on the robot. Moreover, the adjustment of the robot’s trajectories, velocities, and the accelerations will not be done online, first they have to be evaluated, in accordance with the patient bio-medical parameters, and after that a change decision will be made.

Regarding the integrity and safety of patient data with whom the RoboTeleRehab system interacts, in a world where cybercrime and data infringement have dramatically increased, it has become extremely challenging to keep accurate medical records, while still keeping the sensitive information secure. In order to ensure privacy and data integrity of the patients and medical staff that interacts with RoboTeleRehab system, three ways were considered for ensuring the security of data: ensuring an adequate digital infrastructure, train the medical staff, and regular assessments for data accuracy. How the medical history of a patient is compiled, sorted, and assessed is crucial. How well data are kept in healthcare organizations is determined by the precision with which information is handled. If the framework handling, patient data are competent and efficient, there will be fewer discrepancies in the data, and the information will remain coherent. The best protection a healthcare provider has against hackers and the legal issues that may arise as a result is a solid digital infrastructure. 

The digital infrastructure and the RoboTeleRehab system will only function based on bio-medical sensors and employee-processed inputs. Therefore, it is crucial for healthcare and telerehabilitation entities to have staff that are well-trained and competent in their roles in order to process digital frameworks effectively. Staff members who have access to the database network and handle patient records should receive proper training to reduce the likelihood of human error and ensure that the digital infrastructure processes only genuine information.

Furthermore, healthcare and telerehabilitation providers must regularly conduct evaluations and audits to ensure the data they use are accurate and reliable. This must be done to provide patients the best possible medical attention. The efficient distribution of assets and the proper carrying out of operational activities rely on genuine and precise information. Accurate health records data need constant review. Data integrity can only be achieved if the data are correct. In contrast, false information has the ability to not only damage the hospital’s reputation by reflecting poorly on the quality of care provided to patients, but also to bring about legal action against the institution. Conducting risk assessments on a regular basis will be recommended within RoboTeleRehab system since it helps to reveal emerging issues early on. The likelihood of a problem worsening after it has been detected is reduced when effective actions are taken to address the issue. Problems are easier to manage when they are still relatively minor. If you let it fester for too long, it will become impossible to control. The findings of our investigation validate the system’s key benefits of safety, easy to use, and easy to communicate. After they were given adequate training, five of the study’s seven participants were able to finish the session without the need for on-site support, and six of them agreed or strongly agreed with the statement that “the system may be used without any assistance” However, there was some volunteer criticism regarding usability concerns, and future user-friendliness improvements to the system will support clinical use. To determine if this telerehabilitation system can support cardiac rehabilitation on the home-based approach, the effectiveness of the system should also be evaluated. The fact that the system used in this study was developed utilizing already-available commercial resources integrated with some newly developed ones is a real benefit. Most of the commercially available hardware and software utilized in this study, including PCs, tablet PCs, pulse oximeters, cobots, and videoconferencing software, already exist and are easy to use. The system can be quickly reproduced and deployed because these resources are also readily available. 

More than one study has compared the outcomes of a center-based cardiac rehabilitation (CBCR) program to a home-based cardiac rehabilitation (HBCR) program, including improvements in cardiorespiratory fitness, the quality of life, risk factor management, and death rates. HBCR participants, for example, showed an increase in 6-min walk distance (462 m 74 m vs. 421 m 90 m, *p* = 0.03), higher adherence rates, and an increase in physical fitness when compared to CBCR participants [38,39].

Regarding the difference between the proposed system, RoboTeleRehab, and the exoskeletons, the exoskeleton is an externally operated structure that has joints and connections similar to those of a human being, and it is designed to be used as a prosthetic. Patients wear the exoskeleton, which is equipped with actuators that apply torques to various body parts. Exoskeleton actuators supply much of the power needed to execute a job, while the person provides control signals. The individual becomes an integral part of the exoskeleton and exerts a force proportional to his or her new role in the system [17,40] The main difference between the two types of robotic systems used for rehabilitation is that exoskeletons cannot be used at home without qualified supervision, while the system proposed in this paper is intended to be used at home.

Patients with heart failure who participated in a 12-week tele-rehabilitation HBCR program showed no significant improvement in the 6-min walk distance from CBCR at the conclusion of the program [38,39]. Secondarily, findings on patient contentment, quality of life, and muscle strength likewise indicated that the two groups were equivalent [1]. Participants in the CopenHeartRFA study selected between HBCR and CBCR after either atrial fibrillation ablation or heart valve surgery. There were no significant differences between the two groups in terms of gains in peak oxygen uptake, stationary cycle power, and 6-min walk improvement. The baseline physical performance and health of HBCR patients was better than that of CBCR patients [40]. No differences in the groups’ METS on exertion or recovery rate in the first minute following exercise were seen in a small (n = 28), randomized investigation comparing HBCR with CBCR [41]. The AHA/ACC recently investigated HBCR and came to the conclusion that the outcomes of HBCR were equivalent to those of CBCR in terms of functional ability, health-related quality of life, and risk factor reduction [42,43].

Another component of HBCR worth discussing is whether it increases cardiac patients’ adherence and motivation to CR. On the one hand, in a recently published study on 136 cardiac patients, Spindler et al. observed an initial increase in autonomous motivation in the HBCR, which did not persist overtime (12 months). The results indicate that HBCR is comparable to CBCR with regards to motivation, psychological distress, and quality of life. Moreover, these findings suggest that HBCR may not be associated with a greater risk of psychological distress compared to conventional, on-site rehabilitation [44]. On the other hand, two systematic reviews seem to incline towards a higher acceptability of TR as compared cu CBCR. First of all, Owen et al. [45] identified five studies which measured adherence and found that it was higher in patients with HBCR as compared cu CBCR [46,47,48,49,50,51]. One of these studies, using a wrist heart rate monitor showed that the overall recorded rate of completion of the intervention was high (91%), despite concerns about technical problems [48]. The authors observed that older people are willing to engage in the use of new technologies, with a high training adherence of approximately 85% [48]. Lunde et al. measured adherence in the intervention group only, finding that it was ‘high’, with 71% of patients completing all tasks [50]. Secondly, Naghmeh Niknejad et al. [51] published another systematic review referring to adherence and motivation increase following TR programs. The authors identified a total of 32 of 133 studies referring to acceptance and adherence to the rehabilitation programs in different pathologies. The results were conflicting [51]. Finally, Dinesen B et al. proposed Web portal ActiveHeart.dk and the electronic rehabilitation (e-rehabilitation) plan as tools for health education and coordinating rehabilitation goals, in the rehabilitation process cardiac patients [52]. They found that HBTR motivated the patients to integrate rehabilitation activities into their work schedule and everyday life and made them feel like unique individuals. The patients associated HBTR program with a heightened sense of security and autonomy, navigation between active involvement in the rehabilitation process, being an equal partner, and not pushing the patient too hard [52]. Even though the studies rendered conflicting results, with some claiming a very high level of adherence and others observing a decrease in adherence and motivation over time, we must agree that telerehabilitation have a great potential to improve cardiac rehabilitation. Further studies are needed to establish its real impact on cardiac patients.

This research has a few limitations. Although this work and the tests showed that this telerehabilitation system could be feasible, they can still be influenced by the environment because of the internet connection and the lack of knowledge in the field of IT and robotics. These results, however, were really good in a reliable internet environment with a Wi-Fi connection. A level of internet speed that is reliable enough to make video calls should, however, be adequate to give the same quality of intervention as in this study since the system is designed to limit the effect of an unstable internet connection by preinstalling the video software on the user PC. Another limitation of this research is the reduced number of volunteers on which these preliminary tests were done. These preliminary tests helped us to demonstrate the technical feasibility of the system, and we are going to carry out new extensive tests on patients to demonstrate the medical feasibility of the system. Future efforts will concentrate on enhancing aspects regarding the easiness of the RoboTeleRehab system, setup, and remote control. Additionally, a risk analysis will be carried out in a future paper for identifying the associated hazards for the RoboTeleRehab system.

## 5. Conclusions

This work presented the preliminary design and alpha version of a new home-based cardiac telerehabilitation system enhanced by a robotic and Virtual Reality (VR) system—RoboTeleRehab. The RoboTeleRehab system include (A) a home-based telerehabilitation system for the patient and (B) a telemonitoring system for the clinician. The designed home-based cardiac telemonitoring and telerehabilitation system (RoboTeleRehab) aims to aid patients in restoring motor function after a serious cardiac event. It is challenging for the patient to sustain motivation for therapeutic compliance during unsupervised therapy at home. By enhancing the safety, comfort, attractiveness, and enjoyment of the workouts, a collaborative robot (cobot) and virtual reality (VR) module can deliver the required incentive. This helps the therapist to increase the patients’ motivation, engagement, and enjoyment throughout their high-intensity, repeated training sessions.

The main element of novelty that this research brings is the fact that the patient who needs long-term cardiac rehabilitation will relieve the hospitals and will be motivated to continue the rehabilitation therapy at their homes by being in contact with the specialist through the RoboTeleRehab system. Moreover, the integration of the collaborative robot as a component of the rehabilitation system will cause the cardiac patient to be more responsible and motivated; the patient will perceive the hardware side of the RoboTeleRehab system as their direct connection with the medical staff, which will give him/her increased confidence and motivation in the success of the therapy. Moreover, in many families in Romania, elderly people with chronic medical problems (e.g., cardiac) find it very difficult to go to a rehabilitation center or to accept strangers, even medical staff, in their houses; therefore, such a rehabilitation system would be very suitable and an absolute novelty for the Romanian rehabilitation market.

The findings of this study should spur more research on telerehabilitation’s use in clinical settings.

## Figures and Tables

**Table 1:** The subjects’ characteristics and baseline measures.

Variable	Value
Age (years) mean ± SD	45 ± 3
Male (%)	66.6
High education level (%)	100
Body mass index (kg/mp)	32.2
Medical history	
Hypertension (%)	33.3
Obesity (%)	66.6
High cholesterol (%)	33.3

**Table 2:** Results of modified telerehabilitation questionnaire.

Questions	Satisfaction
I would use the system again	4.17
I am satisfied with the quality of the system	4.33
The system is easy to use	4.67
I feel comfortable using the system	4.67
The communication with the trainer was easy	4.50
I can easily understand how to move	4.83
I feel safe performing the exercise	4.67
The used device is appropriate for performing the exercise program	4.50
Telecommunication with the trainer during the exercise was helpful.	4.50
The VR system was helpful in performing the exercises	4.83

Answers scale: 5—strongly agree, 4—agree, 3—undecided, 2—disagree, 1—strongly disagree.

## Data Availability

The data presented in this study are available on request from the corresponding author.
